# Unravelling the Role of Metabolites for Detecting Physiological State of Wild Animals: European Rabbit’s (*Oryctolagus cuniculus*) Case

**DOI:** 10.3390/ani12223225

**Published:** 2022-11-21

**Authors:** Pablo Jesús Marín-García, Lola Llobat, Carlos Rouco, Juan Antonio Aguayo-Adán, Torben Larsen, Maria Cambra-López, Enrique Blas, Juan José Pascual Amorós

**Affiliations:** 1 Department of Animal Production and Health, Veterinary Public Health and Food Science and Technology (PASAPTA), Facultad de Veterinaria, Universidad Cardenal Herrera-CEU, CEU Universities, 46113 Valencia, Spain; 2 Ecology Area, Faculty of Science, University of Cordoba, 14071 Cordoba, Spain; 3 Sociedad, Ecología y Gestión del Medio Ambiente, UCO-IESA, Unidad Asociada al CSIC, 14071 Cordoba, Spain; 4 Department of Animal Science, Aarhus University, DK-8830 Tjele, Denmark; 5 Institute for Animal Science and Technology, Universitat Politècnica de València, Camino de Vera s/n, 46022 Valencia, Spain

**Keywords:** metabolite, biomarker, European rabbit, conservation, ecological nutrition

## Abstract

**Simple Summary:**

The main aim of this work was to know the possible potential of certain metabolites as biomarkers of the European wild rabbit to deepen the biological knowledge of this species and complement specific conservation programs. The main finding of our paper is that, regardless of the weight of the animals and their reproductive state, females show greater feed intake capacity than males, and their metabolism is affected. Furthermore, different reproductive stages are related to different metabolic phenotypes, metabolic behaviors, and metabolic profiles. There are indications of better optimization of resources by females, and evidence of the importance of some metabolites in the reproductive fitness of the species.

**Abstract:**

European wild rabbit (*Oryctolagus cuniculus*) has been defined as a keystone species in the Mediterranean ecosystem. Rabbits have been classed as “endangered” by the IUCN within their native range. In this sense, animal nutrition may play a fundamental and limiting role in the conservation of wild species. The overarching goal of ecological nutrition is to unravel the extensive web of nutritional links that direct animals in their interactions with their ecological environments. The main aim of this work was to evaluate the effect of different feed intake, geographic location, animal sex, and reproductive stage on glucose, non-esterified fatty acids (NEFA), and plasmatic urea nitrogen (PUN), albumin, glutamate, and total protein metabolites. Additionally, we examined the potential of these metabolites as biomarkers. Full stomach contents and blood samples were collected from European wild rabbits (*n* = 89) for the analysis of the metabolites described above. Our work shows that the levels of these metabolites are affected by the sex of the animals, as well as by their reproductive stage (glucose, NEFA and albumin). There were signs of better optimisation of resources by females than by other groups of animals. These data may be interesting in the study of nutritional components that could be affecting physiological state of this species.

## 1. Introduction

The European wild rabbit is considered a threatened species due to the outbreak of different diseases, such as myxomatosis and rabbit haemorrhagic disease, and habitat deterioration and/or fragmentation [1,2]. Consequently, European rabbit mortality values are considerably high, estimated at around 55% only in adults due to rabbit haemorrhagic disease [3], and this species has recently been classified as “endangered” by the International Union for Conservation of Nature (IUCN) [4]. European wild rabbit (*Oryctolagus cuniculus*) is defined as a keystone species [5] because it plays a relevant ecological role in the Mediterranean ecosystem [6,7].

The loss of keystone species is critical to ecosystem structure and functioning, and their introduction constitutes a critical point in the ecological restoration of degraded ecosystems [8]. In this context, animal nutrition plays a fundamental and limiting role in the recovery of these species [9,10]. The overarching goal of ecological nutrition is to unravel the extensive web of nutritional links that drives animals in their interactions with their ecological and social environments [11,12]. Furthermore, it can potentially contribute to multiple research fields [13] such as conservation physiology [14].

Ecological nutrition can use molecular analytical tools to address the management of threatened wildlife [15,16]. Knowing the metabolic phenotype diversity and its relationship with diet type and animal feeding patterns can help increase the understanding of how nutrient availability and use affects the populations of these animals [17]. In addition, delving into the metabolic phenotyping of the different reproductive stages of wild animals can provide us with further information at a biological level. To know the role of different metabolites in different reproductive stages of wild rabbits, and the relevance of these metabolites in the reproductive fitness of this species, is essential to improve recovery, adaptation, and conservation plans for this species, which is key to the preservation of many carnivore species, including species with currently low effective population numbers, such as the Iberian lynx. Therefore, our hypothesis was that some nutritional metabolites could serve as potential biomarkers to determine the feeding level, as well as the reproductive stage and the fitness of the species, by providing information at the biological level.

The main aim of this work was to evaluate the effect of geographic location, animal sex, and reproductive stage on certain metabolites. Additionally, the potential of these metabolites as biomarkers of the physiological state has been studied. 

## 2. Materials and Methods

### 2.1. Animals and Sampling

A total of 89 European wild rabbits were used in this experiment. Animals were free until the moment being of killed from hunters, and were under normal environmental conditions and feeding (Preserve 1—39°31′25.1″ N; 1°21′31.9″ W, *n* = 20; Preserve 2—39°35′37.8″ N; 1°16′44.1″ W, *n* = 25; Preserve 3—38°56′24.6″ N; 0°23′10.3″ W, *n* = 19; and Preserve 4—38°56′23.4″ N; 0°22′46.7″ W, *n* = 25). Full stomach contents and blood were recorded for each animal in spring during morning hours. For each one, a record was made of the sex (male/female), age (young/adult), reproductive stage (for males, non-breeding and in heat; for females, non-breeding, pregnant, and lactating), state of perirenal thickening, weight, length, the day the sample was taken, and its location.

Then, the digestive contents of each animal were extracted and weighed to calculate the full stomach contents weight. Blood samples were taken from the thoracic cavity (1 mL in EDTA vials) and immediately centrifuged for 5 min at 700 G, and the supernatant plasma was extracted. Plasma and full stomach contents were stored frozen (−20 °C) until further analysis.

### 2.2. Chemical Analysis of Blood Nutritional Metabolites

Here, NEFAs (non-esterified fatty acids) were determined using the Wako, NEFA C ACS-ACOD assay method. Analyses were performed using an ADVIA 1800 ^®^Chemistry System autoanalyzer (Siemens Medical Solutions, Tarrytown, NY 10591, USA). 

The PUN (plasmatic urea nitrogen) determination was performed using a commercial kit (Urea/BUN-Color, BioSystems S.A., Barcelona, Spain). The samples were defrosted and tempered, after which 1 μL was pipetted into test tubes.

Blood plasma glucose, albumin, and total protein were determined according to standard procedures (Siemens Diagnostics^®^, Erlangen, Germany, Clinical Methods for ADVIA 1800). 

Glutamate was determined according to Larsen and Fernandéz (2017) [18].

### 2.3. Statistical Analysis

All analyzed traits were fitted to a normal distribution. Least square means were compared by a *t*-test. Metabolites were analysed as dependent variables using a CLM model from SAS [19]. An analysis of the effects of age and sex, animal age (young *versus* adult male and female), and the interaction of these main fixed effects has been included. Additionally, an analysis of the effects of reproductive stage, sex (male and female), reproductive stage (non-breeding males and females, lactating females, pregnant females, and males in heat) and their interactions has also been included. 

The representation of nutritional metabolites obtained with each rabbits’ weight/length was fitted by linear regression with a regression (REG) procedure from SAS [19]. Weight and length of rabbits were not significant when used as covariates.

## 3. Results

The main values of the traits analyzed in this work are summarized in Table 1. We can observe a high variability, since the coefficient of variation for metabolites ranges from 17–70% and is around 60% for the full stomach contents measurement.

Consequently, these data were analyzed per group, considering the effect of geographic location, animal sex, and reproductive stage in rabbits subjected to different feeding patterns. Following this procedure, we determined the effects of age, sex, and reproductive stage. No significant differences were observed between the different geographical areas in any of the parameters analyzed.

### 3.1. Effects of Age and Sex

**Figure 1:**
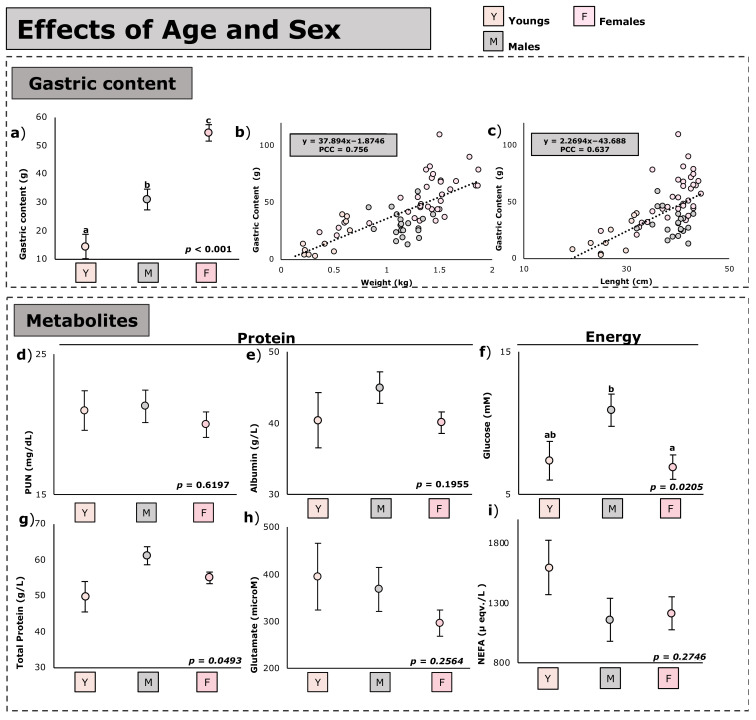
Effect of age and sex on the full stomach contents and nutritional metabolites of *Oryctolagus cuniculus* (*n* = 89). Least square means ± standard errors, (**a**–**c**) Least square means in the same graph with no common superscripts differ significantly at *p* < 0.05 (**a**,**d**–**i**). Relationship between digestive contents weight and biometric measures, i.e., animal body weight (Figure 2b) and animal length (**c**); PCC, Pearson correlation coefficient; NEFA, non-esterified fatty acids; PUN, plasma urea nitrogen.

The effect of age and sex on the nutritional metabolites and the animal’s full stomach contents is shown in Figure 1. 

As regards full stomach contents, three groups of animals (young, males, and females) showed distinct full stomach contents (*p* < 0.001), with the lowest found in young animals (on average −63%; *p* < 0.001 than the adults), followed by males, with the highest being in the females (+76% than males; *p* < 0.001) (Figure 1a). The weight of the full stomach contents was positively correlated with both the weight (Pearson coefficient = 0.75; Figure 1b) and length (Pearson coefficient = 0.64; Figure 1c) of the individuals. 

As regards nutritional metabolites (Figure 1d–i), despite these differences in full stomach contents, there were no differences in the blood metabolites between the three groups of animals (young, male, and females) except for glucose levels. For glucose, females showed lower levels (−63%; *p* = 0.0182) compared to males; young animals presented intermediate values. Finally, marginal statistical difference was found in total protein; a higher quantity was found in males than in the young and females (Figure 1g). 

### 3.2. Effects of Reproductive Stage

**Figure 2:**
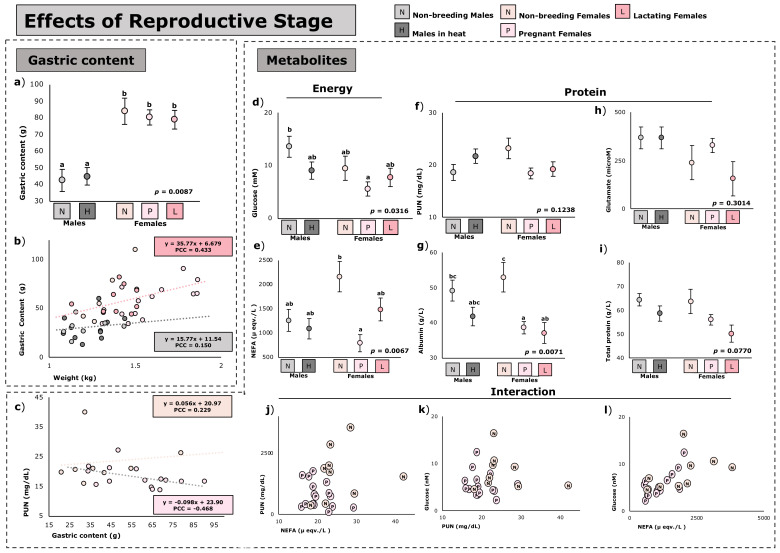
Effect of reproductive stage on the full stomach contents and metabolism of *Oryctolagus cuniculus* (*n* = 62). Least square means ± standard errors, (**a**–**c**); least square means in the same graph with no common superscripts differ significantly at *p* < 0.05 (**a**, **d**–**i**). Relationship between digestive contents with weight and the tendency of males and females (**b**). Relationship between plasmatic urea nitrogen (PUN) with digestive contents, and the tendency of non-breeding females and pregnant females (Figure 2c). Here, PCC is Pearson correlation coefficient. The relationship between animal’s PUN and non-esterified fatty acids (NEFA) (**j**) or with glucose (**k**), and the relationship between glucose and NEFA (**l**) in non-breeding females and pregnant females.

The effect of reproductive stage on nutritional metabolites and the animal’s full stomach contents is shown in Figure 2. Regardless of their reproductive stage, females showed higher (*p* < 0.05) full stomach contents compared with non-breeding males or males in heat (Figure 2a). However, when correlating the full stomach contents with the weight of the animals, females always have a higher intake than males, regardless of their weight (Figure 2b), showing the different tendencies between males and females. 

As regards nutritional metabolites, glucose levels were higher (+142%; *p* = 0.0145) in non-breeding males compared to those of pregnant females, with the rest showing intermediate values (Figure 2d). The NEFA and albumin levels were higher (+171%; *p* = 0.0041 for NEFA and +37%; *p* = 0.0351 for albumin) in non-breeding females compared with pregnant females, while the rest generally presented intermediate values (Figure 2e and g, respectively). Albumin levels were also higher (+42%; *p* = 0.0390) in non-breeding females compared to lactating females. There were no differences in the other blood metabolites amongst animals in different reproductive stages. 

Figure 2c compares the correlation between PUN levels and full stomach contents. As can be seen, there is a different slope between non-breeding females and pregnant females. While there is no clear trend in non-breeding animals, pregnant animals showed lower PUN levels as full stomach contents increased (*p* < 0.05).

Finally, to see whether there are differences in the metabolism of animals at different productive stages, Figure 2j–l lists the individual values of glucose, NEFA, and PUN of non-breeding and pregnant females. As can be seen from these same figures, there is a different grouping between these groups, which would indicate a different metabolic profile.

## 4. Discussion

The main aim of this work was to determine the potential of certain nutritional metabolites as biomarkers applied for the conservation of the European wild rabbit (*Oryctolagus cuniculus*) and their relationship with certain biological traits. Full stomach contents could be determined using an estimate of previous feed intake [20,21,22,23,24,25]. Next, the differences observed between the physiological stages are developed.

The different sexes and reproductive stages also affected the analysed variables. The higher full stomach contents observed in females (+76%) than in males was similar (+72%) to that obtained by Joseph (1909). The reproductive stage also affected the estimation of the ingestion performed in wild animals by Cooke (2014), [26] who observed a higher intake in lactating females (+48%) compared to males. Unravelling the relationship between the nutrition and reproductive stage is critical, as the present samples were obtained during a “reproductive peak” period [27]. Although studies should be carried out to address the feed intake of these animals to a greater extent, it seems that females have a large feed intake capacity. Other authors have shown the ability of females to modulate their feed intake and their metabolic profile according to their nutritional requirements [28]. These authors explained that when reproductive attempts are made, ovarian hormones play a major role in the changes in the ingestion, partitioning, and utilisation of metabolic fuels. Therefore, for an adequate interpretation of the metabolite results, it is important to consider the previous feed intake of the animals (since at different intakes the nutritional use may be different, even though the animals show the same metabolic profile). Moreover, a lower ingestion in males comparted to females could be explained by the reproductive peak because, in this period, the males are more interested in looking for receptive females rather that eat and perform other activities.

In this context, blood metabolite levels could be related to previous feed intake [29]. This is the case for the PUN where, under controlled conditions, the higher the protein intake, the higher the PUN [30]. The absence of some differences in the metabolites analysed between males and females with a significantly higher intake by the females would indicate the possibility of different feeding or resource acquisition methods between these two groups. There is a studied relationship between the availability of energetic nutrients and animal reproduction, approaching the concept of the mechanistic field of reproductive ecology [31,32]. Wade et al. (1992) [28] concluded that reproductive physiology and behaviours are sensitive to the availability of oxidisable metabolic fuels, and this could partly explain the metabolic changes observed; further studies are needed to determine these interactions. The differences observed between groups in energy and nutritional metabolites are described below.

The energetic and protein metabolites showed a clear difference between the reproductive status of the females. Changes in various blood plasma metabolites confirm the intense modification of metabolism in pregnancy [33] and in the growing period [34]. The low circulating levels of glucose and albumin in this period suggest either that body energy stores are depleted by pregnancy or that no energy is available for body tissue deposition [35]. These data are consistent with the NEFA, as non-reproductive females showed higher levels of NEFA than pregnant ones, indicating a short-time mobilisation of adipose tissue [36,37,38]. In this context, the lower NEFA levels of pregnant females could be indicating a lower expenditure of body reserves, compared to non-reproductive females, which could indicate a relationship between these metabolites and the reproduction of this species. Therefore, different metabolic profiles can be observed when comparing females. Although in some of the metabolites analysed there are no significant differences in any metabolite between different reproductive stages, the metabolic behaviour may be different. This is, for example, the case for the PUN, where we observed that, unlike non-breeding females, pregnant females show a tendency towards decreased urea levels when their intake increases, which would give an indication of a more efficient use of resources when nutritional requirements increase in the reproductive season. 

Concrete, energetic metabolites (Glucose and NEFA) were also affected by the physiological state of the animals. However, in this case, protein metabolites and albumin were not affected by the reproductive stage. Knowing biomarkers that are related to reproductive stages could be useful in specific conservation programs, for example in ecosystem management. Including endemic plant conservation strategies is necessary to increase the populations of European wild rabbit and, in turn, the resilience of the Mediterranean ecosystem [39,40].

This work supports the theory that nutrition plays an important role in the reproductive state and, therefore, in the fitness of the species. Our study shows that conservation physiology and nutritional ecology are both integrative sciences that share the fundamental aim of understanding the patterns, mechanisms, and consequences of animal responses to changing environments [14]. Although some work has already been carried out, further work is, however, necessary regarding the knowledge of the metabolic pathways, including integrated genomic, transcriptomic, and metabolomic approaches [41], leading to results with practical consequences that are specifically required to upgrade animal conservation [42].

## 5. Conclusions

The main conclusions drawn from our work are that nutritional metabolites show potential as biomarkers in European wild rabbit (*Oryctolagus cuniculus*) conservation. These conclusions are as follows: (i) Regardless of their weight and their reproductive state, females show greater feed intake capacity than males and their metabolism is affected. Animals of different reproductive stages have different metabolic phenotypes, and metabolic behaviours, and their metabolic profiles allow the differentiation of reproductive stages. There are indications of better optimisation of resources by females. (ii) Glucose, NEFA, and albumins showed potential as biomarkers of animals’ physiological states. Although recently the potential of nutritional metabolites has been demonstrated as an indicator of the level of previous intake [43], further studies are necessary to improve the knowledge of metabolic pathways with potential application as biomarkers, also using integrated OMICs sciences, to upgrade animal conservation.

## Figures and Tables

**Table 1:** Metabolites values (least square means ± standard errors) of the experimental population, obtained from blood samples of European wild rabbits (*Oryctolagus cuniculus*).

Metabolites Analyzed ^1^	Range	Values	Coefficient of Variation (%)
PUN (mg/dL)	12.95–40.00	20.5 ± 0.63	26.0
NEFA (µ eqv./L)	105–3600	1272 ± 99.6	64.6
Glucose (mM)	2.16–30.8	8.16 ± 0.64	63.3
Albumin (g/L)	25.2–57.1	41.5 ± 1.21	16.6
Total protein (g/L)	42.7–71.3	56.3 ± 1.41	14.2
Glutamate (microM)	74–589	323 ± 38.6	38.6
Full stomach contents weight (g)	2.17–109.8	39.2 ± 2.78	59.4

^1^ Here, PUN: is plasma urea nitrogen; NEFA is non-esterified fatty acids.

## Data Availability

The datasets of the current study are available from the corresponding author upon reasonable request.
